# Investigation of oxidant and antioxidant levels in patients with psoriasis

**DOI:** 10.3906/sag-1807-257

**Published:** 2019-08-08

**Authors:** Okan KIZILYEL, Necmettin AKDENİZ, Mahmut Sami METİN*, Ömer Faruk ELMAS

**Affiliations:** 1 Department of Dermatology, Erciş State Hospital, Van Turkey; 2 Department of Dermatology, Faculty of Medicine, İstanbul Medeniyet University, İstanbul Turkey; 3 Department of Dermatology, Batman Medicalpark Hospital, Batman Turkey; 4 Department of Dermatology, Faculty of Medicine, Ahi Evran University, Kırşehir Turkey

**Keywords:** Psoriasis, total oxidant status, total antioxidant status, malondialdehyde, 8-hydroxy 2’-deoxyguanosine

## Abstract

**Background/aim:**

Psoriasis is an immune-mediated chronic inflammatory skin disease that is seen in 1%–3% of the population. It is characterized by symmetrical papulosquamous lesions on the scalp, knees, elbows, sacral region, and extensor surfaces of the extremities. Psoriasis affects both sexes equally. It is thought that reactive oxygen species have an important role in inflammatory skin diseases, especially in psoriasis. There are few studies investigating serum oxidant-antioxidant levels in psoriasis. In this study, we aimed to investigate serum oxidant and antioxidant levels in psoriasis and their effects on its pathogenesis.

**Materials and methods:**

Included in this study were 50 patients with psoriasis who had not been treated with any systemic medication and 45 healthy volunteers (control group). The total oxidant status (TOS), total antioxidant status (TAS), malondialdehyde (MDA), and 8-hydroxy 2’-deoxyguanosine (8H2D) were studied via venous blood sampling. The parameters were measured spectrophotometrically. The study was approved by the Local Ethics Committee of the Atatürk University Faculty of Medicine.

**Results:**

The mean ages of the patients and control group were 32.48 (±14.45) and 35.64 (±17.40) years, respectively. Of the patients, 23 were male and 27 were female. Of the healthy volunteers, 20 were male and 25 were female. The mean disease duration was 8.77 (±6.90) years. The mean Psoriasis Area and Severity Index (PASI) score was 11.41 (±9.62). The mean TOS levels of the patient and control groups were 63.12 (±33.23) and 4.50 (±9.74), respectively. This difference was statistically significant (P = 0.00). The mean TAS levels of the patient and control groups were 3.15 (±0.70) and 3.16 (±0.44), respectively, without any statistically significant difference. The mean MDA levels in the patient and control groups were 14.84 (±6.66) and 12.77 (±4.87), respectively, without any statistically significant difference. The mean 8H2D levels of the patient and control groups were 16,781.2 (±5918.95) and 15,276.13 (±6084.95), respectively. This difference was also not statistically significant. There was no correlation between PASI scores and the above-mentioned parameters.

**Conclusion:**

In the present study, the TOS levels showed a significant statistical difference between the psoriasis and control groups. This finding supports the effect of the oxidant system in the pathogenesis of psoriasis. This was the first study to investigate MDA, TOS, TAS, and 8H2D levels together in patients with psoriasis. More studies are needed to clearly understand the relationship between psoriasis and the oxidant-antioxidant system.

## 1. Introduction

Psoriasis is a chronic inflammatory disease characterized by erythematous scaly papules and plaques. A prevalence of 1%–3% was found in different populations [1]. Lesions are usually localized symmetrically on the scalp, sacral region, and extensor surfaces of the extremities [2]. The disease affects both sexes equally. It may be seen at any age. Psoriasis has been defined as a systemic disease or a metabolic syndrome because of its relation to some systemic comorbid diseases [3]. Nowadays, it is thought that the oxidant and antioxidant systems have a crucial role in the pathogenesis of the disease [4]. When the literature was reviewed, there were few studies investigating the oxidant and antioxidant systems in relation to psoriasis, and the results of these studies were controversial [5,6]. We aimed to understand the pathogenesis of psoriasis better by investigating oxidant and antioxidant parameters. To our knowledge, this was the first study investigating malondialdehyde (MDA), total oxidant status (TOS), total antioxidant status (TAS), and 8-hydroxy 2’-deoxyguanosine (8H2D) levels in patients with psoriasis. 

## 2. Materials and methods

Included in this study were 50 patients and 45 age- and sex-matched healthy volunteers. Patients receiving systemic treatments and pregnant patients were excluded. The age, sex, clinical history, dermatologic findings, and Psoriasis Area and Severity Index (PASI) score were recorded for each patient. Blood samples were left at 25 °C for 30 min without adding any anticoagulant. Next, they were centrifuged at 2000 rpm for 15 min at 4 °C. Serum samples were frozen at –80 °C. The TOS, TAS, MDA, and 8H2D levels were studied for each patient and volunteer. The parameters were measured spectrophotometrically, all of which were statistically compared between the patients and volunteers. The study was approved by the Local Ethics Committee of the Atatürk University Faculty of Medicine.

### 2.1. Malondialdehyde

For the colorimetric standard of MDA, 8 standard samples were prepared. First, 100 µL of sodium dodecyl sulfate was added to 100 µL serum. The mixture was then added to the bottom of Color Regain mixture and centrifuged at 1600 rpm. Next, 150 µL of each sample was taken and placed on the plate for colorimetric measurement.

### 2.2. 8-Hydroxy 2’-deoxyguanosine

The samples were diluted 2 times. The EIA standard was prepared. First, 100 µL of the standard sample was diluted with 900 µL of ultrapure water. The solution concentration was 30 ng/mL. A total of 8 tubes were prepared for the standard. Then 900 µL of EIA buffer was added to tube 1, and 500 µL of EIA buffer was added to tubes 2–8. Next, 100 µL of bulk was added to tube 1. Following that, 400 µL of sample was transferred from tube 1 to tube 2 and a serial dilution was performed for the other tubes. The samples were covered with plastic film and incubated at 4 °C for 18 h. To each tube, 200 µL of Ellman reagent and 500 µL of tracer were added. The samples were measured at 410 nm. 

### 2.3. Total antioxidant status

For the preparation of the CAL standard, 30 µL sample was used. For reagent 1, 500 µL of sample and 500 µL of standard were mixed. For reagent 2, 75 µL of sample and 75 µL of standard were mixed. The samples were measured spectrophotometrically at 660 nm and 37 °C.

### 2.4. Total oxidant status

For reagent 1, 500 µL of sample and 500 µL of standard were mixed. For reagent 2, 25 µL of sample and 25 µL of standard were mixed. The samples were measured spectrophotometrically at 660 nm and 37 °C.

### 2.5. Statistical evaluation

The Student t-test and chi-square test were used to evaluate differences between the groups. Correlation analyses were performed via Pearson’s correlation test. P < 0.05 was accepted as statistically significant.

## 3. Results

The mean age was 32.48 (±14.45) and 35.64 (±17.40) years for the patients and the control group, respectively. Of the patients, 23 were male and 27 were female. In the control group, 20 were male and 25 were female. No significant relationship was found between the patients and the control group in terms of age (P = 0.34) or sex (P = 0.88). The mean disease duration was 8.77 (±6.90) years. Of the patients, 13 had only psoriatic skin lesions without itching, 35 had itchy psoriatic skin lesions, and 2 had itchy psoriatic skin lesions and arthralgia. There was a history of arthritis in 5 patients. The mean PASI score was 11.41 (±9.62). The PASI scores were classified as mild (0–9 points) in 29 patients, moderate (10–19 points) in 10 patients, and severe (≥20 points) in 11 patients. Only 3 patients showed nail involvement. The demographic and clinical parameters are provided in Table 1.

**Table 1:** Demographic and clinical parameters in patients and control group.

	Patients	Control group
Mean age	32.48 years	35.64 years
Sex	Male: 23, female: 27	Male: 20, female: 25
Mean disease duration	8.77 years	-
Patients with only skin lesions	13	-
Patients with skin lesions and itching	35	-
Patients with skin lesions, itching, and arthralgia	2	-
PASI score	Mild (0–9 points): 29 patientsModerate (10–19 points): 10 patientsSevere (≥20 points): 11 patients	-

The mean TOS level was 63.12 (±33.23) for the patients and 14.50 (±9.74) for the control group. The TOS levels were significantly higher for patients when compared to the control group (P < 0.00). The mean TAS level was 3.15 (±0.70) for the patients and 3.16 (±0.44) for the control group. The TAS levels showed no significant difference between the patients and the control group (P = 0.91). The mean MDA level was 14.84 (±6.66) for the patients and 12.77 (±4.87) for the control group. The MDA levels also showed no significant difference between the patients and the control group (P = 0.11). The mean 8H2D level was 16,781.2 (±5918.95) for the patients and 15,276.13 (±6084.95) for the control group. There was no significant difference between the patients and the control group for 8H2D values (P = 0.26). There was also no correlation between the PASI scores and TAS (P = 0.85), MDA (P = 0.18), 8H2D (P = 0.96), or TOS (P = 0.67) levels. The mean levels of MDA, TOS, TAS, and 8H2D in the patients and the control group are given in Table 2.

**Table 2:** Mean MDA, TOS, TAS, and 8H2D levels in patients and control group.

	Patients	Control group	P-value
Mean MDA	14.84	12.77	P = 0.11
Mean TOS	63.12	14.50	P < 0.00*
Mean TAS	3.15	3.16	P = 0.91
Mean 8H2D	16,781.2	15,276.13	P = 0.26

*Statistically significant.

## 4. Discussion

When the literature was reviewed,****there were some studies suggesting that oxidative stress plays an important role in the pathogenesis of multifactorial skin diseases like psoriasis, atopic dermatitis, and vitiligo. These diseases showed increased levels of oxidant molecules and decreased levels of antioxidant molecules as a result of lipid peroxidation [7]. It has been considered that an increased number of neutrophils causes increased levels of reactive oxygen species and releases proteolytic enzymes that destroy the surrounding tissue. Increased levels of reactive oxygen species cause increased levels of phospholipase A2 and lipid peroxidation. As a result, cyclic adenosine monophosphate (cAMP) is deactivated but cyclic guanosine monophosphate (cGMP) is activated. Decreasing the cAMP/cGMP ratio causes epidermal hyperproliferation in psoriasis [8]. As a natural response, the antioxidant system is activated when the oxidant system is activated. However, this response is insufficient in psoriasis [9] and reactive oxygen species seem to be related to the activation of psoriatic lesions. It has been shown that reactive oxygen species have chemotactic effects on neutrophils [10]. There have been some studies investigating the oxidant and antioxidant systems in psoriasis with controversial results. Kural et al. found that the MDA and lipid peroxide levels were increased but the catalase, superoxide dismutase (SOD), glutathione peroxidase, and TAS levels were decreased in patients when compared to the control group [6]. Kökçam et al. and Wozniak et al. also found that MDA levels were increased in patients with psoriasis [11,12]. In the present study, no significant difference was observed in MDA levels between the patients and the control group. Baz et al. found that MDA and SOD levels were increased and the antioxidant potential was decreased in patients with psoriasis [4]. In this study, there was no correlation between PASI scores and MDA, SOD, or antioxidant potential. Emre et al. found that TAS levels were decreased and TOS levels were increased in psoriasis and there was no relation between PASI scores and TAS or TOS [13]. The present study also found that TOS levels were increased but TAS levels were not decreased in the patient group and there was no correlation between PASI scores and oxidant-antioxidant parameters. Kadam et al. [14] and Gabr et al. [15] found that MDA levels were increased and TAS levels were decreased in patients with psoriasis. Kaur et al. found that TAS levels were decreased in their patients [16]. In our study, there was no significant difference in TAS and MDA levels between the two groups.

As mentioned above, there have been some studies investigating MDA levels in psoriasis and these studies showed controversial results. Yıldırım et al. studied MDA levels in both blood and tissue samples and found no statistically significant increase in MDA levels in the blood samples. However, they showed that there was a significant increase in the tissue samples [7]. In a study by Severin et al., there was no significant difference in TAS levels [5]. Usta et al. detected no significant changes in TOS and TAS levels for patients with psoriasis [17]. In the present study, increased TOS levels were detected in the patient group. However, no significant difference was observed in TAS levels between the two groups. With regards to 8H2D, there have been a few studies investigating urine levels of 8H2D, but to the best of our knowledge, there have not been any studies investigating 8H2D levels in blood samples. Hence, this was the first study investigating blood levels of 8H2D in psoriasis. In our study, there was no significant difference in 8H2D levels between the groups. Similarly, Toker et al. also found that the lipid, MDA, and TAS levels were not significantly different in psoriasis cases when compared to a control group; however, the paraoxonase and arylesterase levels were significantly higher [18]. Houshang et al. found that MDA levels were increased and paraoxonase levels were decreased in patients with psoriasis [19]. The clinical type of the disease, demographic features, treatments, and distribution of the lesions could explain these controversial results. Different test methods may also cause different results. Oxidant and antioxidant molecules can only pass in a certain amount from tissue to serum. Therefore, the serum levels of the parameters may not fully reflect the tissue levels. The use of tissue biopsy materials may provide results that are more accurate.

It is thought that the balance of the oxidant and antioxidant systems is disturbed in psoriasis. Studies investigating oxidant and antioxidant levels in psoriasis have shown controversial results. In the present study, there was no significant difference in TAS, MDA, and 8H2D levels between the patients and the control group; however, TOS levels were significantly higher in the patient group. Therefore, we suggest that it is better to choose TOS in order to determine oxidative stress in patients with psoriasis. An important feature of the present research is that this was the first study investigating blood levels of 8H2D in psoriasis. The most important limitation of our study was the limited number of patients and controls. Therefore, it is obvious that further studies with a larger sample size are needed to clearly reveal the role of the oxidant-antioxidant system in psoriasis.
